# Radiation dose escalation based on FDG-PET driven dose painting by numbers in oropharyngeal squamous cell carcinoma: a dosimetric comparison between TomoTherapy-HA and RapidArc

**DOI:** 10.1186/s13014-017-0793-0

**Published:** 2017-03-23

**Authors:** Sarah Differding, Edmond Sterpin, Nicolas Hermand, Bianca Vanstraelen, Sandra Nuyts, Nathalie de Patoul, Jean-Marc Denis, John Aldo Lee, Vincent Grégoire

**Affiliations:** 1 Department of Radiation Oncology, and Center for Molecular Imaging, Oncology and Radiotherapy (MIRO), Université catholique de Louvain, Institut de Recherche Expérimentale et Clinique (IREC), Brussels, Belgium; 2 Department of Oncology, Experimental Radiation Oncology, KU Leuven - University of Leuven, Leuven, Belgium; 3 Department of Radiation Oncology, University Hospitals Leuven, Leuven Cancer Institute, Leuven, Belgium; 4 Department of Radiation Oncology, St-Luc University Hospital, Avenue Hippocrate 10, B-1200 Bruxelles, Belgium

## Abstract

**Purpose:**

Validation of dose escalation through FDG-PET dose painting (DP) for oropharyngeal squamous cell carcinoma (SCC) requires randomized clinical trials with large sample size, potentially involving different treatment planning and delivery systems. As a first step of a joint clinical study of DP, a planning comparison was performed between Tomotherapy HiArt® (HT) and Varian RapidArc® (RA).

**Methods:**

The planning study was conducted on five patients with oropharyngeal SCC. Elective and therapeutic CTVs were delineated based on anatomic information, and the respective PTVs (CTVs + 4 mm) were prescribed a dose of 56 (PTV_56_) and 70 Gy (PTV_70_). A gradient-based method was used to delineate automatically the external contours of the FDG-PET volume (GTV_PET_). Variation of the FDG uptake within the GTV_PET_ was linearly converted into a prescription between 70 and 86 Gy. A dilation of the voxel-by-voxel prescription of 2.5 mm was applied to account for geometric errors in dose delivery (PTV_PET_). The study was divided in two planning phases aiming at maximizing target coverage (phase I) and lowering doses to OAR (phase II). A Quality-Volume Histogram (QVH) assessed conformity with the DP prescription inside the PTV_PET_.

**Results:**

In phase I, for both HT and RA, all plans achieved comparable target coverage for PTV_56_ and PTV_70_, respecting the planning objectives. A median value of 99.9 and 97.2% of all voxels in the PTV_PET_ received at least 95% of the prescribed dose for RA and HT, respectively. A median value of 0.0% and 3.7% of the voxels in the PTV_PET_ received 105% or more of prescribed dose for RA and HT, respectively. In phase II, no significant differences were found in OAR sparing. Median treatment times were 13.7 min for HT and 5 min for RA.

**Conclusions:**

Both HT and RA can generate similar dose distributions for FDG-PET based dose escalation and dose painting in oropharyngeal SCC patients.

**Electronic supplementary material:**

The online version of this article (doi:10.1186/s13014-017-0793-0) contains supplementary material, which is available to authorized users.

## Introduction

After concomitant chemo-radiotherapy for locally advanced squamous cell carcinoma of the head and neck (HNSCC), still 20 to 30% of patients suffer from loco-regional recurrences, typically within the gross tumor volume (GTV) [1], motivating local dose escalation. Over the last decade, the introduction of intensity modulated radiation therapy (IMRT) creating larger dose gradients between target volumes and the surrounding normal tissues has opened an avenue for radiation dose escalation. However, the dose can only be increased to some extent; indeed increased late toxicity has been observed with increased dose prescription [2]. This observation led to the concept of image-driven dose painting, where the dose is prescribed, delivered and possibly increased non uniformly within target volumes based on maps of detrimental biological factors such as tumor hypoxia, tumor metabolism, cell proliferation [3–6]. In dose painting by numbers (DPBN), the dose is prescribed and possibly escalated as a function of the voxel intensity of a given molecular imaging modality [3].

Clinical validation of a DPBN strategy requires the conduction of randomized clinical trials with a large number of patients treated in several centers with most likely different treatment planning and delivery systems. According to the website “clinicaltrial.gov”, there are at the time of writing four ongoing randomized phase II trials about dose escalation in HNSCC patients. But while most protocols are based on a uniform dose escalation inside a predefined volume (ClinicalTrials.gov identifier: NCT01212354, NCT02352792, NCT02089204) only one study is recruiting at present for a non uniform dose escalation based on DPBN (Ghent University Hospital in Belgium, ClinicalTrials.gov identifier: NCT01341535). This study uses in-house treatment planning systems (TPS), and its methodology may not be straightforward to transpose in commercial TPS.

Over the last few years, the feasibility of generating DPBN plans with commercial TPS e.g. Helical TomoTherapy (HT) and Varian RapidArc (RA) has also been demonstrated, and several planning studies have been conducted on HNSCC cases [7–9]. However, there is no head-to-head comparison of the capabilities of both solutions in the context of dose escalation based on FDG-PET DPBN, where a very precise voxel-by-voxel dose distribution has to be obtained. Hence, the question arises whether both treatment systems can achieve similar performance and thus be used within the same clinical trial of DPBN.

As a first step on the way towards a joint clinical trial, this study carries out a dosimetric comparison of dose painting plans with a dose escalation from 70 to 86 Gy inside the GTV delineated in FDG-PET images. The aim was to evaluate the capabilities of both HT and RA systems to plan and deliver accurately a given complex non-uniform dose prescription, and to challenge both systems in extreme clinical configurations. Dose heterogeneity pushes treatment-planning systems to their limits because steep and controlled dose gradients are needed.

## Material and methods

### Patient selection and image acquisition

Five patients with stage IV (TNM 2007) oropharyngeal SCC treated with concomitant chemo- or cetuximab-radiotherapy were selected. All patients had a minimal tumor diameter of 3 cm. Information about the exact tumor location, the staging and the tumor volume are presented in the Additional file 1.

Patients underwent a contrast-enhanced CT and ^18^F-FDG-PET on a combined PET/CT camera (Gemini TF, Philips Medical system, Cleveland, OH, USA). Contrast-enhanced CT scans were performed using a slice thickness of 2 mm, a reconstruction interval of 2 mm and a pitch of 0.8. Axial images were reconstructed using a matrix of 512 × 512 pixels with a size of 0.52 × 0.52 × 1 mm^3^. The three-dimensional PET images were acquired with an axial field of view of 155 mm (two bed positions centered on the primary tumor), a matrix of 288 × 288 pixels with a size of 2 × 2 × 2 mm^3^. Acquisitions were performed 90 min after the injection of 281–304 MBq of ^18^F-FDG. The PET images were reconstructed with the 3D line of response, time-of-flight (TOF) blob-based ordered subset expectation maximization (OSEM) algorithm from Philips with 3 iterations and 33 subsets [10]. The resolution of the PET images measured in the center of the field of view (FOV) with a point source in attenuating material led to a full width at half maximum (FWHM) of 6.5 mm.

More details about image acquisition and segmentation have already been published [8].

### Target volume, Organ at Risk and dose-volume constraints

#### Delineation of the PET-based target volumes

A gradient-based method was used to delineate automatically the external contours of the primary tumor FDG-PET GTV (GTV_PET_) [11]. Quantitative conversion of the PET signal into a heterogeneous dose prescription from 70 Gy to 86 Gy was performed using a linear relationship between the median and maximal ^18^F-FDG uptake values in the GTV_PET_. Robustness against geometrical uncertainties was ensured with the methodology introduced by Sterpin et al. [12]. The PTV_PET_, which was used for dose prescription, was obtained accordingly by dilating the voxel-by-voxel prescription for the GTV_PET_ by 2.5 mm to account for systematic errors, while random errors could be neglected. In order to guide the optimizer to approach the voxel-by-voxel prescription, seven equidistant sub-levels were defined inside the PTV_PET_ allowing grouping the voxels into defined sub-volumes. The threshold of each sub-level, corresponding to a percentage of the maximum FDG uptake into the PTV_PET_ was then converted into a dose prescription, as a percentage of the maximal dose increment of 16 Gy. This threshold value was set to the minimal dose constraint in the TPS. As the FDG uptake voxel distribution within each sub-level is known to be heterogeneous, the maximum dose constraint for the i^th^ level equaled the minimum dose constraint of level i + 1.

#### Clinical target volumes and Organ At Risk

Clinical target volumes (CTV) and the nearby organs at risk (OAR) were delineated on the planning CT using the CMS® treatment planning software (Elekta Computerized Medical System, version 4.64.00, Stockholm, Sweden) as already described [8]. In short, the so-called therapeutic CTV_70_ (i.e. the CTV with a dose prescription of 70 Gy) was defined as the GTV delineated in the planning CT + 5 mm (both for the primary tumor and for the lymph node metastases), taking into account that bone, cartilages, ligaments and muscles prevent tumor spread. The so-called prophylactic CTV for both the primary tumor (normal tissue at risk of tumor spread surrounding the GTV) and the bilateral elective lymph node areas (delineated according to Grégoire et al. [13, 14]) were united to create the CTV_56_ (i.e. the CTV with a dose prescription of 56 Gy). Last, the PTV_70_ and PTV_56_ were drawn by expanding the CTVs with an isotropic 4 mm margin [15]. A volume defined as a 3 mm thick layer within the patient body contour was excluded from the PTV to avoid skin overdosage. The following OARs were contoured: spinal cord, brainstem, parotid glands, oral cavity, larynx, lower pharyngeal constrictor muscle (PCM), cricopharyngeal muscle, cervical esophagus and mandible. OARs lying (almost) completely within the PTV (i.e. submandibular glands, superior and middle PCM) were not contoured. A planning at risk volume (PRV) was created by adding a 4 mm margin around the spinal cord and the brainstem.

#### Dose-volume constraints

As aforementioned, the PTV_PET_ dose prescription varied linearly with the FDG uptake, from its median value to its maximum. The additional dose volume constraints used are presented in Table 1.

**Table 1:** Dose-volume constraints for the PTV and OAR

Target/Organ at risk	Median absorbed dose (D_50%_) (Gy)	Mean absorbed dose (Gy)	D_near-min_ (D_98%_) (Gy)	D_95%_	D_near-max_ (D_2%_) (Gy)
PTV_56_	56	-	≥90% of prescribed dose	≥95% of prescribed dose	-
PTV_70_	70	-	≥90% of prescribed dose	≥95% of prescribed dose	-
PRV spinal cord	-		-	-	≤50
PRV brainstem	-		-	-	≤50
Contralateral parotid gland	-	≤26	-	-	-
Ipsilateral parotid gland	-	≤30	-	-	-
Oral cavity	-	≤30	-	-	-
Larynx	-	≤40	-	-	-
Lower PCM	-	≤50	-	-	-
Cricopharyngeal muscle	-	≤50	-	-	-
Cervical esophagus	-	≤35	-	-	-
Mandible	-	-	-	-	D_5%_ ≤ 70

*PCM* pharyngeal constrictor muscle

### Design of the planning study

The study was divided into two phases. The first phase aimed at evaluating the ability of both planning and delivery systems to conform the treatment plans to a heterogeneous dose prescription using a set of pre-defined constraints to the OAR. The focus was set on the PTV coverage. To allow the maximum degrees of freedom for target coverage, only four OARs were considered (i.e. ipsilateral and contralateral parotid glands, PRV spinal cord and PRV brainstem). The second phase aimed at evaluating the ability of both systems to plan the heterogeneous dose escalation of 16 Gy inside the PTV_PET_ while lowering the dose to the OARs as much as possible. In this two-step procedure, coverage of the PTV_PET_ could be analyzed before and after possible degradation caused by the dose decrease to OARs.

In each institution, only one operator performed the treatment planning for all patients (SD for HT and NH for RA) under the supervision of a senior physicist. To ensure a fair comparison, for each case, the operators were given the possibility to further improve the plan quality by evaluating what was achieved by the other technique. All plans were considered final after a second round of plan modification and no additional change was allowed thereafter. Clinical acceptability of all plans was checked by two head-and-neck radiation oncologists (VG and SN). The plans were transferred to the commercial software MIM 6.1. (MIMVista corp, Cleveland, Ohio) for visualization and comparison using the same sampling algorithm [16].

### Planning techniques

#### Helical Tomotherapy plans

For HT, treatment planning was performed on a Tomotherapy research planning station version 136.1111.0 with a GPU architecture, with accuracy comparable to the extensively validated CPU algorithm [17, 18]. Based on previous planning optimization, longitudinal field width of 1.05 cm, a maximum modulation factor of 3 and a pitch of 0.215–0.43 were used. These parameters showed to be optimal for DP plans with HT in a previous publication [8]. The dose calculation was performed with the collapsed-cone-convolution algorithm and a grid size set to ‘fine’, which typically corresponds to a 2 × 2 mm^2^ resolution in the transverse plane.

#### RapidArc plans

RA plans were generated for a Varian CLINAC 2100 C/D equipped with the 120-leaf Millennium Multileaf Collimator system, with forty 5-mm central leaf-pairs and twenty 10-mm peripheral leaf-pairs. The plans were optimized using the Progressive Resolution Optimizer (PRO) 10.0.28. After optimization, the final dose distribution was calculated using the Anisotropic Analytical Algorithm (AAA 10.0.28) with a grid size of 1.5 × 1.5 mm^2^.

Optimal plans were obtained for a 6-MV beam and 4 full arcs with collimator rotation of 5°–15° and 75°–85°. The isocenter was centered on the volume of the highest dose prescription. The contribution of each arc to the PTV_PET_ was assured by a minimal jaws opening of 2 cm over the isocenter and a X-jaws opening up to 15 cm, i.r. the maximal leaf-traveling per bank. Collimator angles differed of at least 10° to increase the degree of freedom of the optimizer while remaining close to the typical in-house standard of 2 full arcs with collimator rotation of 10° and 80°. Modulation was pushed to about 280 MU/arc.

Both dose calculation algorithms use conventional “rescaled-to-water” calculation.

### Plan evaluation

Plans were evaluated qualitatively by visual inspection of the dose distribution on each axial CT slice, and quantitatively from the Dose Volume Histograms (DVH) and the Quality Volume Histograms (QVH). For all PTVs but the painted volume, D_50%_ (median dose), D_95%_, D_98%_ (near-minimal dose) and D_2%_ (near-maximal dose) were chosen. Homogeneity of the dose distribution was calculated with the homogeneity index as described in the ICRU 83 report [19]. The degree of conformity of the plans was evaluated through the conformity index, CI_95%_, i.e. the ratio between the patient volume receiving at least 95% of the prescribed dose and the volume of the PTV.

QVHs were used to assess the dose conformity with the non-uniform dose prescription inside the PTV_PET_, as proposed by Vanderstraeten et al. [20]. The Q-value is the ratio between the planned dose and the prescribed dose at each dose voxel inside the dose-painted volume i.e. PTV_PET_. Vanderstraeten et al. [20] also introduced the quality factor (QF) given by ∑_*i*=1…*N*_ |1 - Q_*i*_|/N, where N is the total number of dose points at which Q-values are calculated. Planning objectives were then set as follows: V_Q=0.95_ ≥ 95%, V_Q=1.05_ ≤ 5% and QF ≤ 5% [20, 21]. For QVH calculation, an in-house research platform was used.

Beam-on-times were directly given by the TPS in HT while for RA they were calculated in RT Chart, ARIA (Varian Medical Systems, Palo Alto, CA, USA).

### Quality assurance on the dose delivery (DQA)

DQA on the dose delivery was performed on patient #3. For HT, as mentioned above, treatment planning was performed on a research version of the Tomotherapy TPS. The latter not being connected to the clinical treatment station, DQA could not be performed. In order to bypass this limitation, a new plan meeting the planning constraints of phase II was calculated on the clinical treatment planning station version 5.1.0.4 for patient #3. The treatment plans were delivered on a Tomotherapy HD®.

For both RA and HT, the dose distribution verification consisted of a film measurement (EBT-3 type Gafchromic® films) as well as point dose measurements made with ion chambers (A1SL Exradin miniature Shonka thimble type chamber, Standard Imaging, Inc., Middleton, WI). For RA, these measurements were performed in the arcCHECK phantom (Sun Nuclear Corp, Melbourne FL) used as a simple cylindric phantom [22]. For HT, these measurements were performed in a specially designed solid-water phantom called the cheese phantom [23].

The agreement between the calculated and the measured film dose distributions was evaluated with a global gamma index. Three % / 3 mm was selected as acceptance criteria in the gamma analysis.

### Statistical analysis

Differences in parameters of interest between treatment planning systems were analyzed with the Wilcoxon signed-rank test (SPSS statistical package; version 17.0; SPSS Inc.; Chicago, IL) and p-values lower than 0.05 were considered statistically significant.

## Results

**Fig. 1:**
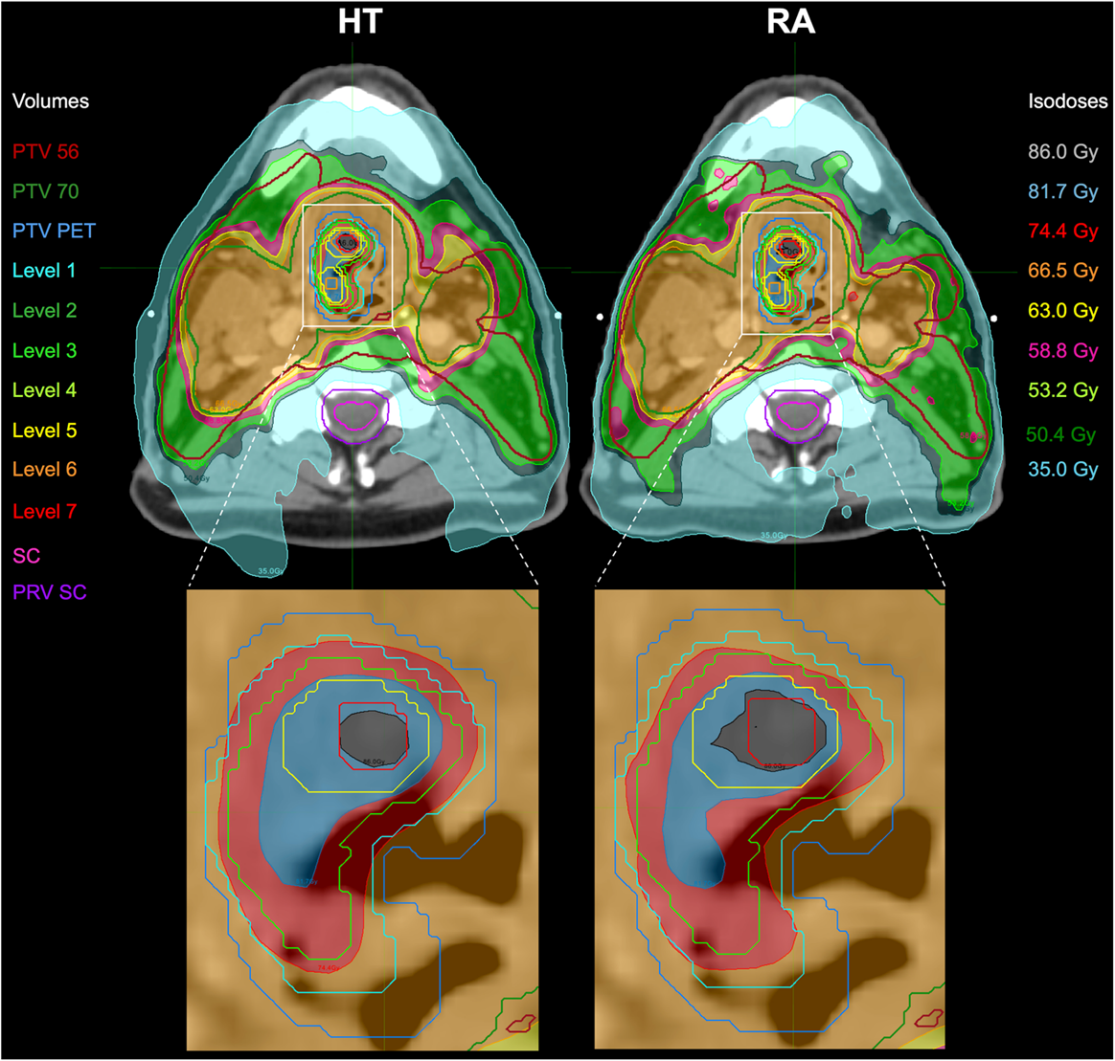
PTV and isodose distribution (planning phase II) for Helical Tomotherapy (HT) and Varian RapidArc (RA) for patient #5. The captions are zooms of the PTV_PET_. Only PET levels 1,3,5 and 7 are depicted to improve the visualization

**Fig. 2:**
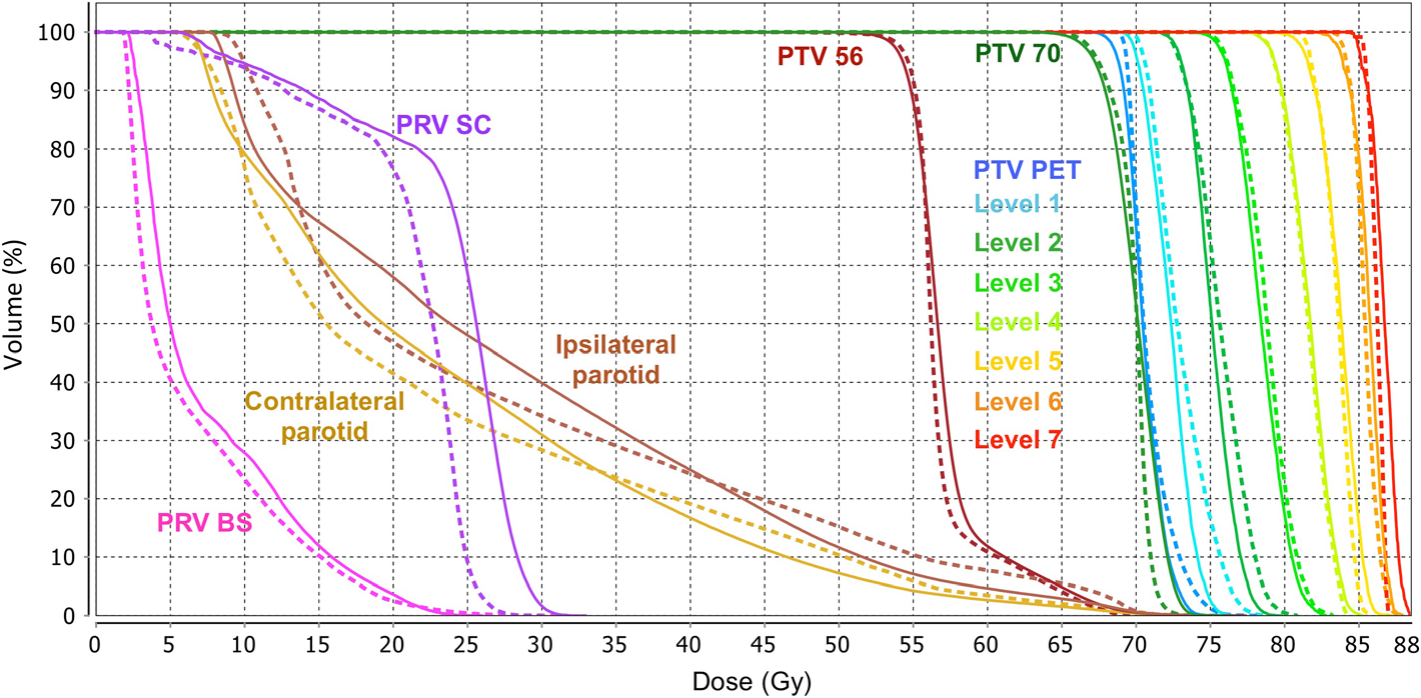
Dose-Volume Histogram (DVH) for the Planning Target Volume (PTV) and the Organ At Risk volume (OAR) for patient #5 in planning phase II. The DVH for the PTVs are represented in a non-overlapping mode. The plain lines are for RapidArc and the dashed lines are for Helical Tomotherapy. (BS: brainstem; SC: spinal cord)

As an example, comparative isodose distribution on axial slices between HT and RA is presented for patient #5 (Fig. 1). The corresponding DVH for PTVs and OARs are displayed in Fig. 2. Similar figures for the 4 other patients are presented in the Additional file 2.

Tables 2 and 3 summarize the quantitative data analysis for PTV and OARs for planning phases I and II, respectively. For planning phase I, the focus was set on the ability of both systems to reach adequate PTV coverage and QVH values. For all patients, the clinical requirements for PTV coverage and OARs sparing are met for both HT and RA. PTV_70_ and PTV_56_ show similar results except for D_2%_ of PTV_70_ and PTV_56_, and for D_50%_ of PTV_56_ that are all statistically significantly smaller for HT. Moreover, HT achieves higher conformity for PTV_56_. No differences are seen on dose homogeneity between HT and RA.

**Table 2:** Comparison between various metrics in planning phase I. The data are the median and range values of the five patients included into the study. PTV_70+PET_ corresponds to the PTV_70_ encompassing the PTV_PET_, while PTV_70_ is the part of the PTV_70_ outside of the PTV_PET_. In a similar way, PTV_56_ is the part of the PTV_56_ outside of the PTV_70_

PHASE I		RapidArc		Helical Tomotherapy		*p*-value
		Median	Min - Max	Median	Min - Max	
QVH indexes	V_Q=0.95_ (%)	99.9	98.7–100.0	97.2	92.7–97.6	0.043
	V_Q=1.05_ (%)	0.0	0.0–0.2	3.7	2.0–4.9	0.043
	QF (%)	1.4	1.2–1.8	1.9	1.8–2.3	0.043
PTV_70+PET_	D_98%_ (Gy)	67.3	67.0–67.8	67.4	66.7–67.8	n.s.
	D_95%_ (Gy)	68.0	67.7–68.4	68.3	67.5–68.5	n.s.
	D_50%_ (Gy)	70.1	70.0–70.4	70.1	69.8–70.3	n.s.
	D_2%_ (Gy)	79.1	74.9–81.3	79.4	76.3–82.2	n.s.
	V_95%_ (%)	99.4	99.1–99.8	99.4	98.3–99.8	n.s.
	HI	0.2	0.1–0.2	0.2	0.1–0.2	n.s.
PTV_70_	D_98%_ (Gy)	67.2	66.9–67.8	67.2	66.6–67.7	n.s
	D_95%_ (Gy)	67.9	67.5–68.3	68.1	67.4–68.4	n.s
	D_50%_ (Gy)	69.9	69.8–70.4	70.0	69.7–70.3	n.s
	D_2%_ (Gy)	72.1	72.0–72.5	71.5	70.8–72.0	0.043
	V_95%_ (%)	99.3	98.9–99.8	99.3	98.1–99.8	n.s
	V_107%_ (%)	0.0	0.0–0.0	0.0	0.0–0.1	n.s
	HI	0.1	0.1–0.1	0.1	0.0–0.1	n.s
PTV_56_	D_98%_ (Gy)	54.1	53.9–54.6	54.6	53.9–54.9	n.s.
	D_95%_ (Gy)	54.6	54.4–54.9	55.0	54.3–55.2	n.s.
	D_50%_ (Gy)	56.6	56.4–57.0	55.9	55.8–56.2	0.043
	D_2%_ (Gy)	68.2	67.4–68.5	68.0	65.9–68.2	0.043
	V_95%_ (%)	99.6	99.2–99.9	99.8	99.5–99.9	n.s.
	V_107%_ (%)	13.8	11.7–21.7	11.5	9.8–20.3	n.s.
	HI	0.2	0.2–0.3	0.2	0.2–0.3	n.s.
PRV SC	D_2%_ (Gy)	47.6	47.1–47.8	47.2	43.0–47.6	-^a^
PRV BS	D_2%_ (Gy)	39.2	34.0–47.2	35.8	25.4–43.4	-^a^
Ipsilateral parotid	D_mean_ (Gy)	28.8	28.5–29.5	28.7	27.1–29.8	-^a^
Contralateral parotid	D_mean_ (Gy)	24.8	23.9–25.1	22.9	21.3–25.2	-^a^
Conformity Index	CI PTV_70_	1.2	1.2–1.2	1.2	1.1–1.2	n.s.
	CI PTV_56_	1.7	1.6–2.0	1.5	1.4–1.8	0.043

^a^no Wilcoxon signed-rank test performed as the dose to OAR had to reach the constraint but was not further optimized in planning phase I

**Table 3:** Comparison between various metrics in planning phase II. The data are the median and range values of the five patients included into the study. PTV_70+PET_ corresponds to the PTV_70_ encompassing the PTV_PET_ while PTV_70_ is the part of the PTV_70_ outside of the PTV_PET_. In a similar way, PTV_56_ is the part of the PTV_56_ outside of the PTV_70_

PHASE II		RapidArc		Helical Tomotherapy		*p*-value
		Median	Min - Max	Median	Min - Max	
QVH indexes	V_Q=0.95_ (%)	99.7	99.4–100.0	95.3	92.2–96.4	0.043
	V_Q=1.05_ (%)	0.2	0.0–0.4	2.6	0.7–6.5	0.043
	QF (%)	1.5	1.3–1.6	1.9	1.8–2.3	0.043
PTV_70+PET_	D_98%_ (Gy)	66.4	65.7–67.0	66.6	66.0–67.0	n.s.
	D_95%_ (Gy)	67.3	66.8–67.9	67.6	67.0–67.7	n.s.
	D_50%_ (Gy)	70.3	70.1–70.5	70.0	69.9–70.3	0.043
	D_2%_ (Gy)	79.7	75.2–81.7	79.6	75.6–81.7	n.s.
	V_95%_ (%)	97.8	96.2–98.8	98.1	96.8–99.0	n.s.
	HI	0.2	0.1–0.2	0.2	0.1–0.2	n.s.
PTV_70_	D_98%_ (Gy)	66.3	65.6–66.5	66.4	65.9–66.7	n.s.
	D_95%_ (Gy)	67.2	66.6–67.5	67.3	66.9–67.5	n.s.
	D_50%_ (Gy)	70.1	69.8–70.3	69.9	69.8–70.1	0.042
	D_2%_ (Gy)	73.0	72.7–73.4	71.6	71.3–71.9	0.043
	V_95%_ (%)	97.6	95.4–98.0	97.7	96.4–98.5	n.s.
	V_107%_ (%)	0.0	0.0–0.1	0.0	0.0–0.0	n.s.
	HI	0.1	0.1–0.1	0.1	0.1–0.1	0.039
PTV_56_	D_98%_ (Gy)	53.6	53.2–53.8	53.9	51.6–54.3	n.s.
	D_95%_ (Gy)	54.4	54.1–54.5	54.6	53.6–54.9	n.s.
	D_50%_ (Gy)	56.7	56.5–57.2	55.9	55.7–56.2	0.043
	D_2%_ (Gy)	67.5	67.0–68.1	65.8	65.7–67.2	0.042
	V_95%_ (%)	98.7	98.0–99.2	99.0	96.1–99.2	n.s.
	V_107%_ (%)	12.0	10.1–20.7	11.0	8.6–14.2	0.043
	HI	0.2	0.2–0.3	0.2	0.2–0.3	n.s.
PRV SC	D_2%_ (Gy)	27.8	24.5–29.9	26.4	24.8–28.5	n.s.
PRV BS	D_2%_ (Gy)	22.5	17.2–24.4	21.3	17.9–23.7	n.s.
Ipsilateral parotid	D_mean_ (Gy)	26.6	24.8–28.9	26.8	25.0–28.3	n.s.
Contralateral parotid	D_mean_ (Gy)	21.6	19.2–23.9	21.0	20.4–24.7	n.s.
Oral cavity	D_mean_ (Gy)	28.5	19.4–42.0	28.2	18.1–44.4	n.s.
Oral cavity minus PTV	D_mean_ (Gy)	18.3	16.6–22.3	19.8	15.8–27.0	n.s.
Lower PCM	D_mean_ (Gy)	38.2	26.0–47.7	37.5	24.0–50.9	n.s.
Cricophar. muscle	D_mean_ (Gy)	23.6	14.9–35.2	22.5	13.8–34.7	n.s.
Cervical esophagus	D_mean_ (Gy)	15.1	12.6–19.7	14.2	10.0–21.4	n.s.
Larynx	D_mean_ (Gy)	46.1	29.3–54.0	49.6	24.2–54.7	n.s.
Larynx minus PTV	D_mean_ (Gy)	21.6	17.3–30.5	23.2	17.3–28.8	n.s.
Mandible	D_5%_ (Gy)	58.5	57.0–65.5	59.2	55.8–68.5	n.s.
Conformity index	CI PTV_70_	1.1	1.0–1.1	1.1	1.0–1.1	n.s.
	CI PTV_56_	1.3	1.3–1.5	1.3	1.2–1.4	n.s.

*PCM* pharyngeal constrictor muscle

**Fig. 3:**
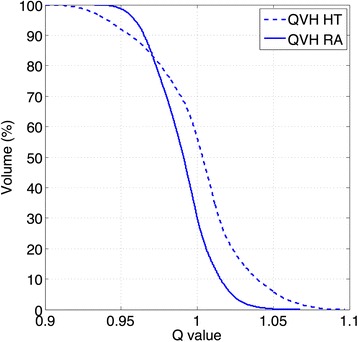
Quality Volume Histogram (QVH) for the PTV_PET_ for patient #5 in planning phase I

Regarding QVH values, QF stays below 2% for both modalities in all patients but patient #5. For this patient’s HT plan, V_Q=0.95_ reaches 92.7% and V_Q=1.05_ reaches 4.9%, while the QF raises up to 2.3% (Fig. 3). Globally, mean QVH values are statistically significantly better for RA than HT. Similar QVHs for the four other patients are provided in Additional file 3. Last, regarding treatment times, median treatment times are 13.7 min for HT and 5 min for RA. Total planning times are estimated to be around 2–3 h for TomoTherapy and around 3–4 h for RapidArc to obtain a dose distribution achieving all planning objectives. If the total time gives advantage to HT, it has to be mentioned that the interaction time between the user and the TPS is shorter with RA, the longest step for each iteration being the final dose calculation where the user doesn’t need to interact with the TPS any longer.

For planning phase II, the constraints are achieved for all OARs with both HT and RA except for the larynx and the oral cavity due to close proximity of the PTV. When this was the case, the dose was lowered as much as possible in the part of the organ outside of the PTV plus a 5 mm-wide region. Regarding PTV coverage, both HT and RA met the constraints with subtle differences between the two systems, few of them reaching statistical significance, but not clinical significance. Regarding the QVH analysis, similar conclusions as for phase I can be drawn. Systematic differences favoring RA were observed.

Last, regarding the QA, a good agreement between the planned and delivered dose distribution was found in patient #3 with gamma pass rates above 96% for the 3%/3 mm criteria for both HT and RA (Additional file 4).

## Discussion

In summary, our study shows that providing the systems are pushed to their upmost limit and cross-checked, both HT and RA can produce similar dose distributions in patients with SCC of the oropharynx using FDG-PET dose painting for dose escalation. The various metrics used for plan comparison were nearly identical; when differences were noticed between the two techniques, they did not reach any level of clinical significance. By “pushed to their upmost limit and cross-checked”, we meant that the objective of our study was not so to identify a “winner” in this planning exercise, but rather to demonstrate that two planning and delivery systems can achieve similar dose distribution in the framework of dose painting for dose escalation, providing adequate settings of the two systems are used. However, this is an initial assessment based on a small sample size and it would be interesting to expand it to a larger number of patients.

To our knowledge, this study presents the first comparison between two rotational IMRT techniques, in the context of dose escalation based on FDG-PET dose painting. HT has already been compared to step-and-shoot IMRT for dose escalation and dose painting plans [24]. A higher degree of conformity was observed with the inhomogeneous dose prescription with HT than with nine fields step-and-shoot IMRT, although the authors concluded that both modalities were clinically suitable for dose painting. On the other hand, several studies comparing rotational IMRT systems without dose escalation in patients with HNSCC have been reported in the literature [25–30]. Most of them found significantly higher normal tissue dose sparing with HT than with RA [25, 28–30]. Regarding target coverage, Stromberger et al. reported only small differences comparing both modalities for unilateral and bilateral simultaneous integrated boost plans [28]. Our study revealed that both rotational techniques generate relatively similar dose distribution regarding both PTV coverage and OAR sparing. However, one should mention that to obtain such conformed plans with RA, we had to use more arcs than what is routinely used in the clinics. The use of four independent arcs that were simultaneously optimized might have allowed the RA’s optimizer to achieve higher target coverage and OAR sparing than in studies where only one or two arcs were used, especially when it turned to compute heterogeneous dose prescriptions such as in dose painting. Such finding has also been reported by Martin et al. who found a significant improvement in plan quality with the use of additional arcs [16]. In terms of conformity, HT achieved a better CI for PTV_56_ than RA. This is consistent with the study of Wiezorek, while Van Gestel et al. reported a better CI for the RA modality [29, 30]. Our results showed that homogeneity in PTV_70_ was higher with HT, confirming results of previous studies [25, 29, 30]. But it should be mentioned that, although statistically significant, such small differences between HT and RA did not reach any significance from a clinical point of view, i.e. that no clinical advantage is expected both from a toxicity or a local tumor control point of view.

The design of a comparative study between different planning and delivery systems impacts on the final results. In our study, each operator had the opportunity to further improve plan quality in a second round after a first comparison was done. The main advantage of such strategy is that we ensure that the physical capabilities of the systems are tested to their limits. However, it does not give information about how would perform an experienced planner on a particular system without having any knowledge of the capabilities of the other system. This chance for a “rebuttal” plan has been found only in one comparison study between arc therapy and HT [31]. Next, the starting point varies between different comparison studies. Most of them take a dataset of previously treated patients and perform a new optimization for each evaluated technique [25–30]. In other studies, however, the delivered treatment plan is taken as reference and a new plan is retrospectively optimized for the other techniques [32, 33]. It remains then questionable how far the initial plans were optimized in comparison to the new plans generated in the frame of a study. Last, the two phases design of our study with a first phase aiming at maximal target coverage with soft OAR constraints and a second one aiming at maximal OAR sparing brings more clarity in the results.

The total planning time was estimated between 2 and 4 h for both planning systems. This is obviously more than what is typically required for a routine homogeneous dose distribution in HNSCC. It should be noticed that HT and RA planning was done by two different operators, who both had to learn how to achieve the best dose distribution for such complex plans. Comparison between the 2 systems is thus difficult, but it looks like a more satisfactory dose distribution was achieved quicker with HT than with RA.

Of special interest to us is the comparison between HT and RA for the non-uniform dose planning. Although both systems were able to achieve clinically acceptable QVH values, a slight advantage was observed for RA. Intrinsic differences between both TPS regarding the optimization process and the leaf sequencing could be a possible explanation to this observation. In particular, RA simulations were performed with a MLC with 120 leaves (5 mm leaf’s width), which resulted in a higher longitudinal resolution than with the HT collimator, which has a smallest jaw width of only 1.05 cm. In the longitudinal direction, the resolution of HT will consequently be limited by the jaw width whereas the resolution of the RA collimator is limited only by the width of the leaves. This could limit the capability of HT of creating very high modulation’s degree. In addition, resolution differences between the TPS of Tomotherapy and Varian could also be responsible for differences in QVH quality. Sub-levels inside the PTV_PET_ are very small and close to each other. A small difference in contour modeling and/or in the interpolation of dose grid data to sample points in these tiny volumes could account for differences in the calculated dose at the voxel level. Furthermore, the QF calculation in itself suffers from the important limitation that values will always be heavily weighted to the DP sub-volumes with small dose increment, due to the larger number of voxels within these larger, peripheral regions.

In these technically complex dose painting plans, as already reported by others in HNSCC patients, delivery with RA clearly proves to be faster than with HT, even though four arcs were used [29, 30]. This is explained by the fact that HT plans had to be performed all the way through with a jaw width of 1.05 cm to allow high conformity in the longitudinal axis, leading to a significant increase in treatment times. An ideal workaround would involve the ability to vary the jaw width of the HT collimator and the speed of the couch movement when it comes to irradiate parts of the PTV that do not require high modulation. Unfortunately, such option is not available in the HT system. Treatment time remains important not only for the patient’s comfort but also because of possible intra-fraction motion of the tumor. Such phenomenon is, however, negligible in oropharyngeal SCC [34].

Last, in this study, the comparison between RA and HT was also performed from a delivery point of view in one patient, and it showed that DPBN plans could be successfully delivered by both techniques.

## Conclusion

In summary, all plans in both groups achieved acceptable target coverage for all included volumes, respecting the planning objectives except for one patient where all QVH objectives could not be met with HT. Such a comparison was necessary to ensure that patients treated with both systems can be accrued in the same multicentric clinical studies. In this context, a phase-I study (ClinicalTrials.gov identifier: NCT02336711) on FDG-PET based dose escalation and dose painting has recently started at the UCL Cliniques universitaires St-Luc and KUL Gasthuisberg universitair ziekenhuis, equipped with HT and RA, respectively.

## Additional files

Additional file 1: illustration of individual contours of investigated patients. (DOCX 1173 kb)

Additional file 2: images of individual dose volume histograms and dose distributions of patients. (DOCX 15022 kb)

Additional file 3: Quality volume histograms of the five patients for planning phase I. (DOCX 116 kb)

Additional file 4: Delivery quality assurance for patient # 3. (DOCX 20 kb)
