# Supplementary material for: Radiation dose escalation based on FDG-PET driven dose painting by numbers in oropharyngeal squamous cell carcinoma: a dosimetric comparison between TomoTherapy-HA and RapidArc
Source: Radiat Oncol. 2017 Mar 23;12:59. doi: 10.1186/s13014-017-0793-0 (PMC5364636; doi:10.1186/s13014-017-0793-0)
Supplement: Supplementary file 1 — illustration of individual contours of investigated patients. (DOCX 1173 kb) [file 13014_2017_793_MOESM1_ESM.docx]

Additional file 1: illustration of individual contours of investigated patients

Figure S1. Patient #1


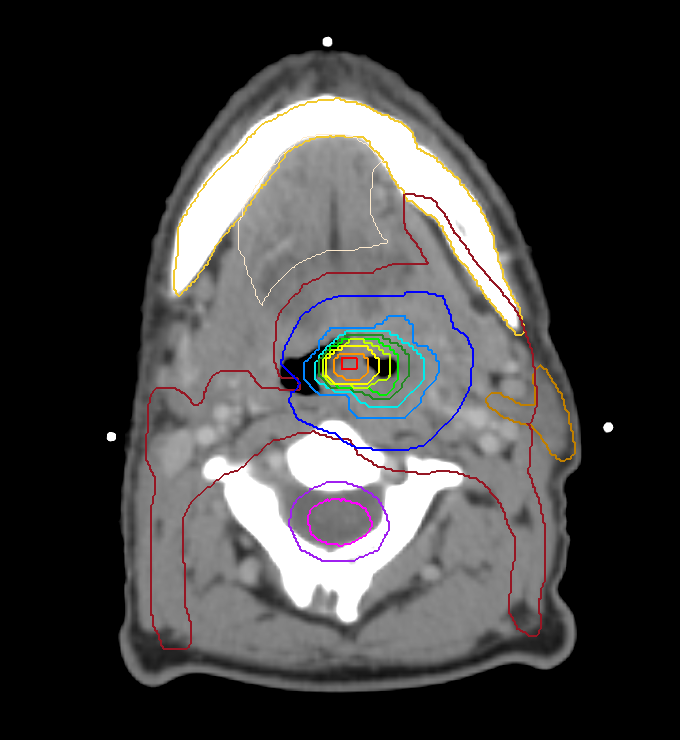


PAR

PRV SC

PTV PET

Level 7

Level 6

Level 5

Level 4

Level 3

Level 2

Level 1

PTV 70

PTV 56

Mandible

Oral cavity

SC

Figure S2. Patient #2


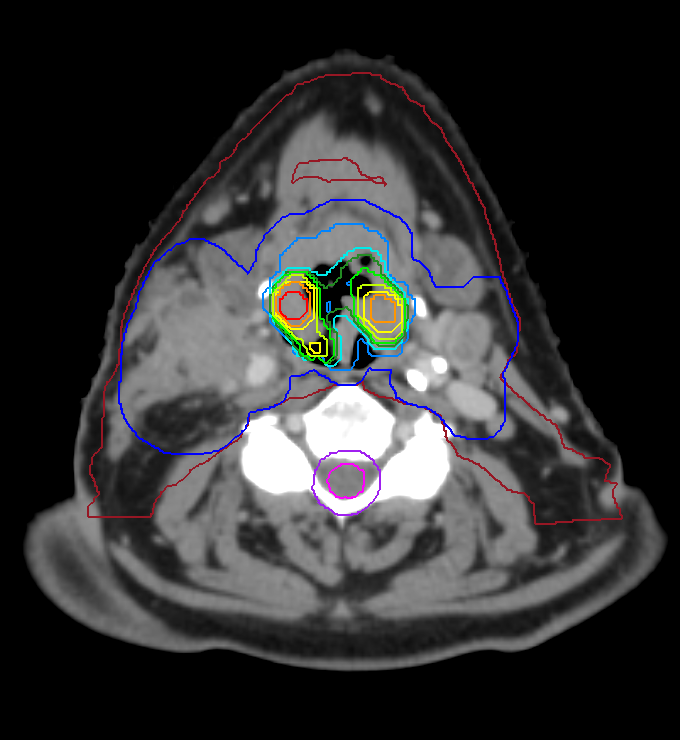


PTV 56

PRV SC

SC

Level 7

Level 6

Level 5

Level 4

Level 3

Level 2

Level 1

PTV PET

PTV 70

Figure S3. Patient #3


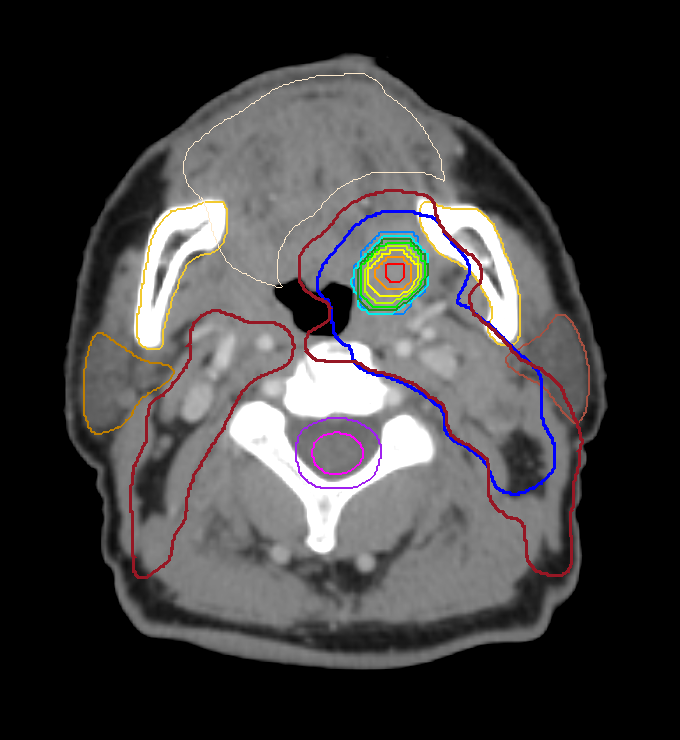


PAR

Level 7

Level 6

Level 5

Level 4

Level 3

Level 2

Level 1

PTV PET

PAR

PRV SC

SC

PTV 70

PTV 56

Oral cavity

Mandible

Figure S4. Patient #4


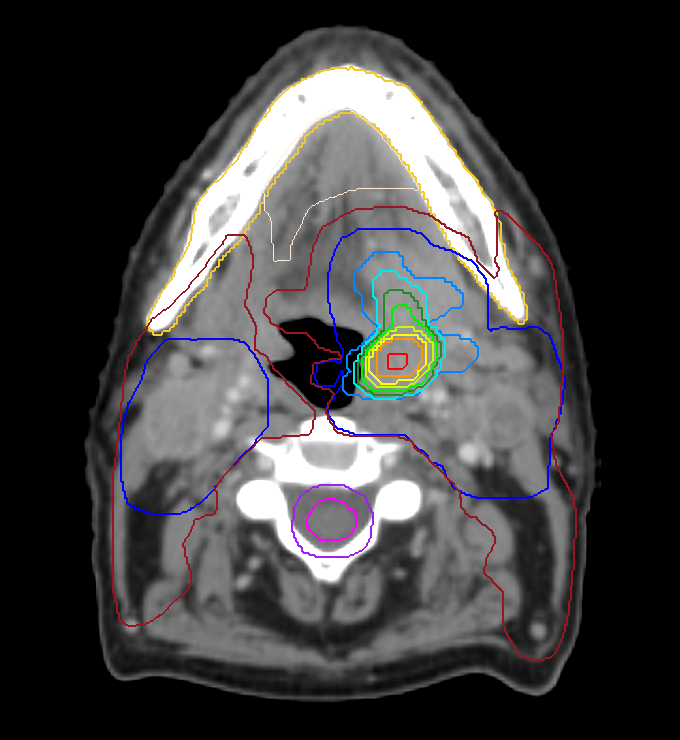


PTV 56

Level 7

Level 6

Level 5

Level 4

Level 3

Level 2

Level 1

PTV PET

Mandible

Oral cavity

PRV SC

SC

PTV 70

Figure S5. Patient #5


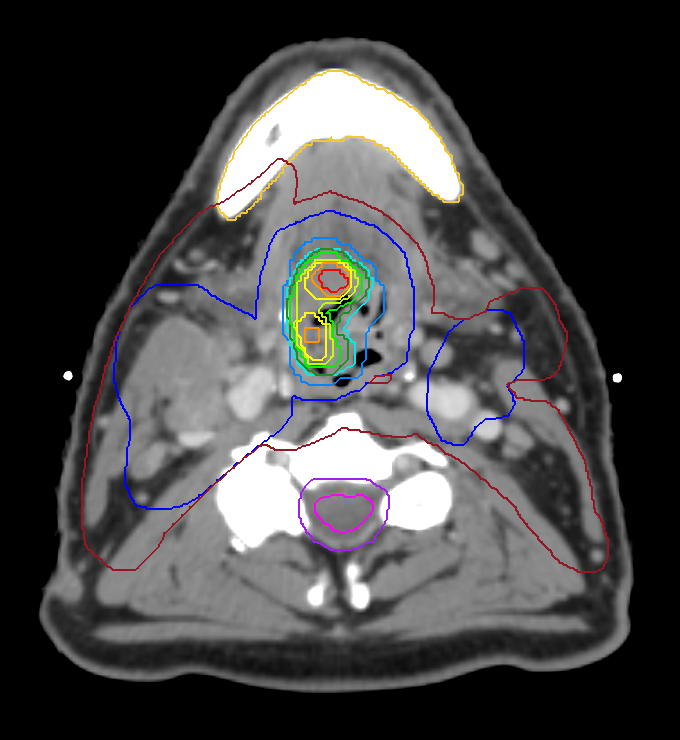


Level 7

Level 6

Level 5

Level 4

Level 3

Level 2

Level 1

PTV PET

PRV SC

SC

PTV 70

PTV 56

Mandible

Legend

PTV 70 = therapeutic planning target volume; PTV 56 = prophylactic planning target volume;

PTV PET = dose painting target volume

SC = spinal cord; PRV SC = planning risk volume of the spinal cord; PAR = parotid gland

| **Pt** | **Location** | **TNM stage** | **Volumes (cm^3^)** | | | | | | | | | |
| --- | --- | --- | --- | --- | --- | --- | --- | --- | --- | --- | --- | --- |
|  |  |  | **PTV_PET_** | **Level** | | | | | | | **PTV_70_** | **PTV_total_** |
|  |  |  |  | **1** | **2** | **3** | **4** | **5** | **6** | **7** |  |  |
| 1 | Lateral pharygeal wall L | T4N0M0 | 36.8 | 19.3 | 14.2 | 9.9 | 6.4 | 3.5 | 1.6 | 0.4 | 98.7 | 482.1 |
| 2 | Vallecula R | T4N2cM0 | 26.4 | 14.2 | 10.4 | 7.2 | 4.6 | 2.6 | 1.1 | 0.3 | 273.6 | 707.6 |
| 3 | Tonsil L | T2N2bM0 | 10.6 | 6.5 | 4.8 | 3.3 | 2.2 | 1.2 | 0.5 | 0.1 | 132.7 | 467.5 |
| 4 | Tonsil L | T4N2cM0 | 38.8 | 20.4 | 14.9 | 10.4 | 6.7 | 3.7 | 1.7 | 0.4 | 216.8 | 702.8 |
| 5 | Base of tongue | T3N2cM0 | 21.7 | 11.8 | 8.7 | 6.0 | 3.9 | 2.1 | 0.9 | 0.2 | 209.2 | 888.5 |

Table S1 Patients characteristics (Pt: patient)
