# Supplementary material for: Radiation dose escalation based on FDG-PET driven dose painting by numbers in oropharyngeal squamous cell carcinoma: a dosimetric comparison between TomoTherapy-HA and RapidArc
Source: Radiat Oncol. 2017 Mar 23;12:59. doi: 10.1186/s13014-017-0793-0 (PMC5364636; doi:10.1186/s13014-017-0793-0)

Additional file 2: images of individual dose volume histograms and dose distributions of patients

Patient #1

Figure S1. Dose-Volume Histogram (DVH) for the Planning Target Volume (PTV) and the Organ At Risk volume (OAR) for patient #1 for the planning phase II. The DVH for the PTVs are represented in a non-overlapping mode. The plain lines are for RapidArc and the dashed lines are for Helical Tomotherapy. (BS: brain stem; SC: spinal cord).


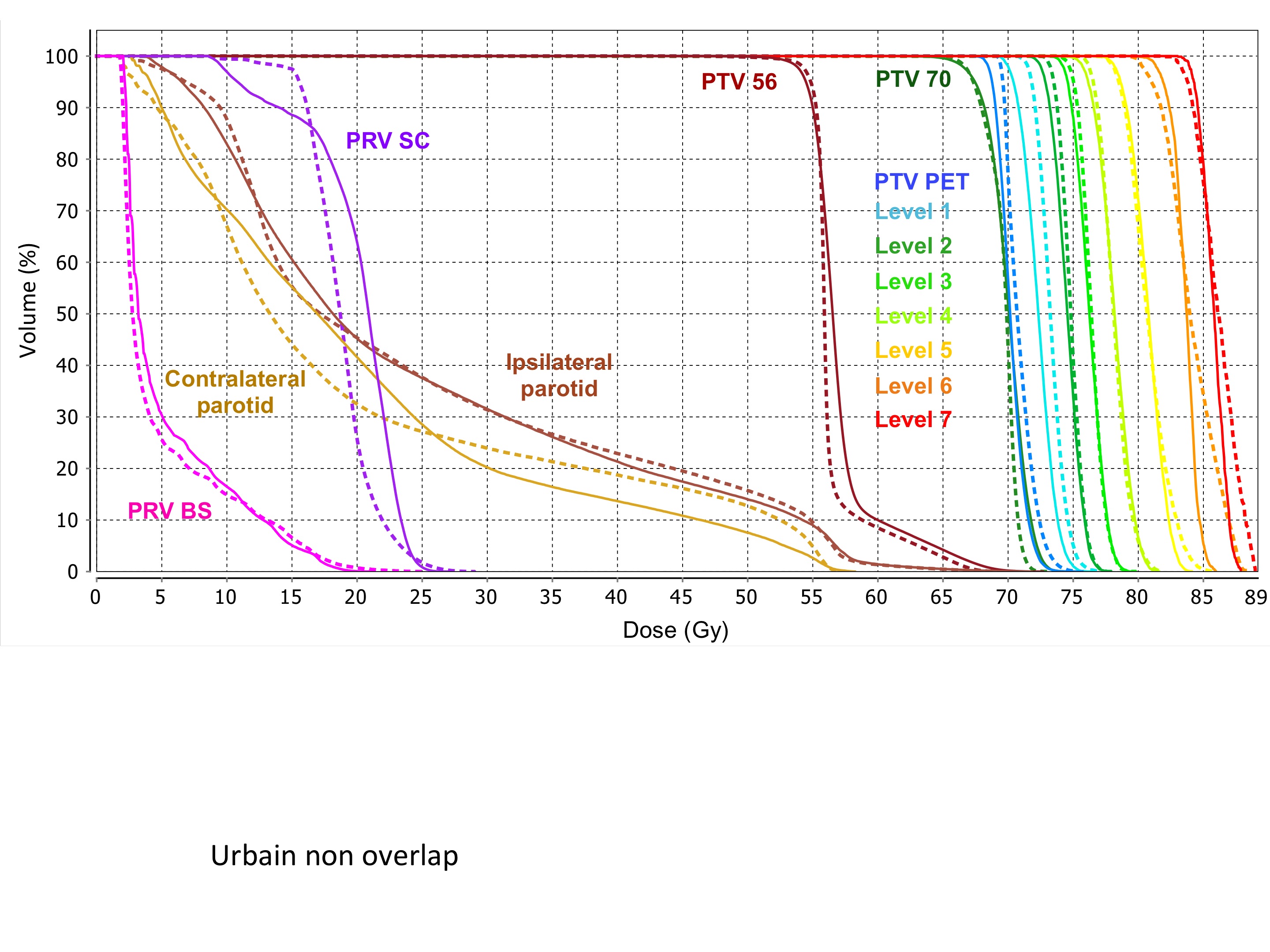


Figure S2. PTV and isodose distribution for Helical Tomotherapy (HT) and Varian RapidArc (RA) for patient #1 for the planning phase II. The captions are zooms of the PTV_PET_.


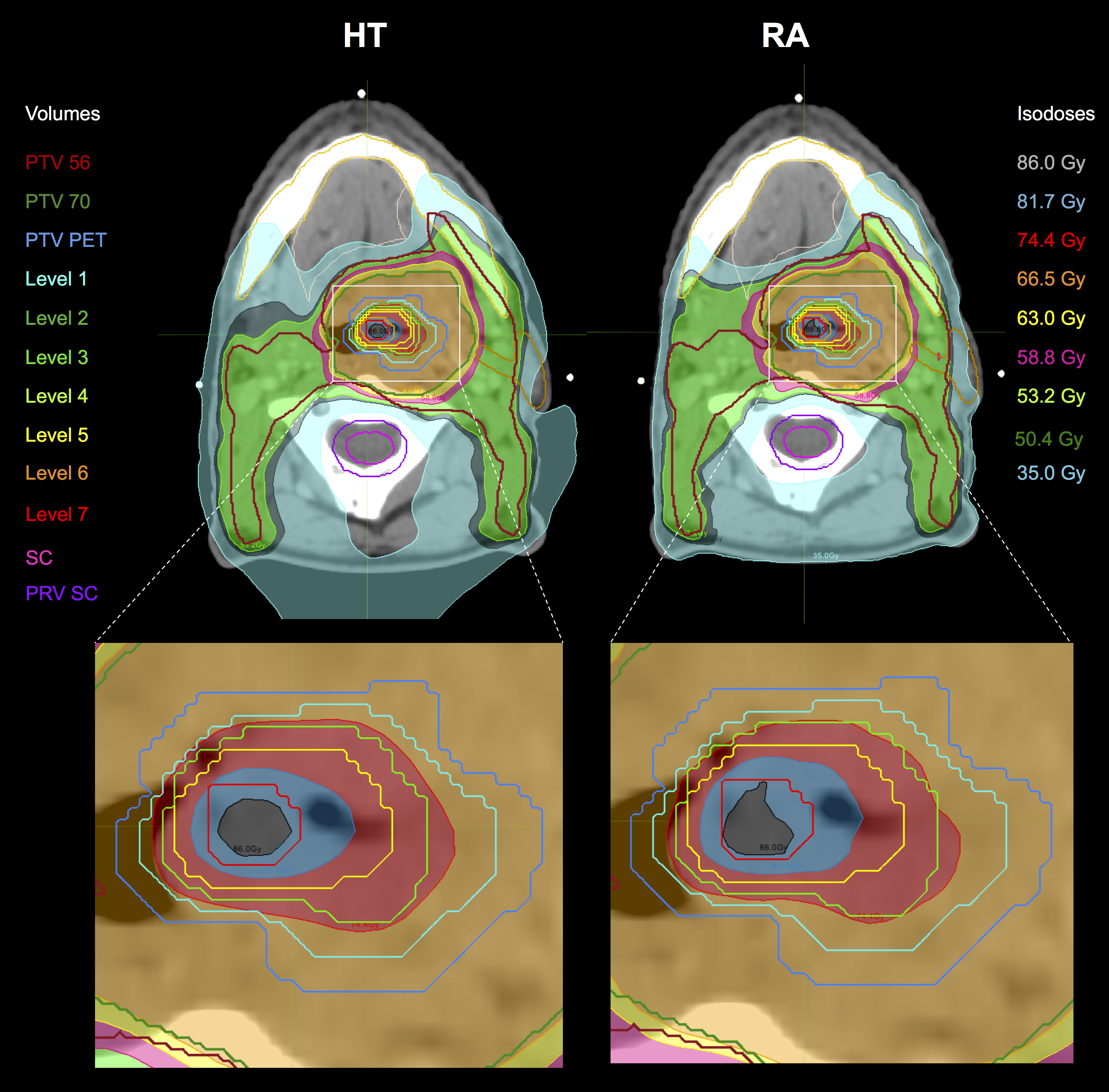


Patient #2

Figure S3. Dose-Volume Histogram (DVH) for the Planning Target Volume (PTV) and the Organ At Risk volume (OAR) for patient #2 for the planning phase II. The DVH for the PTVs are represented in a non-overlapping mode. The plain lines are for RapidArc and the dashed lines are for Helical Tomotherapy. (BS: brain stem; SC: spinal cord).


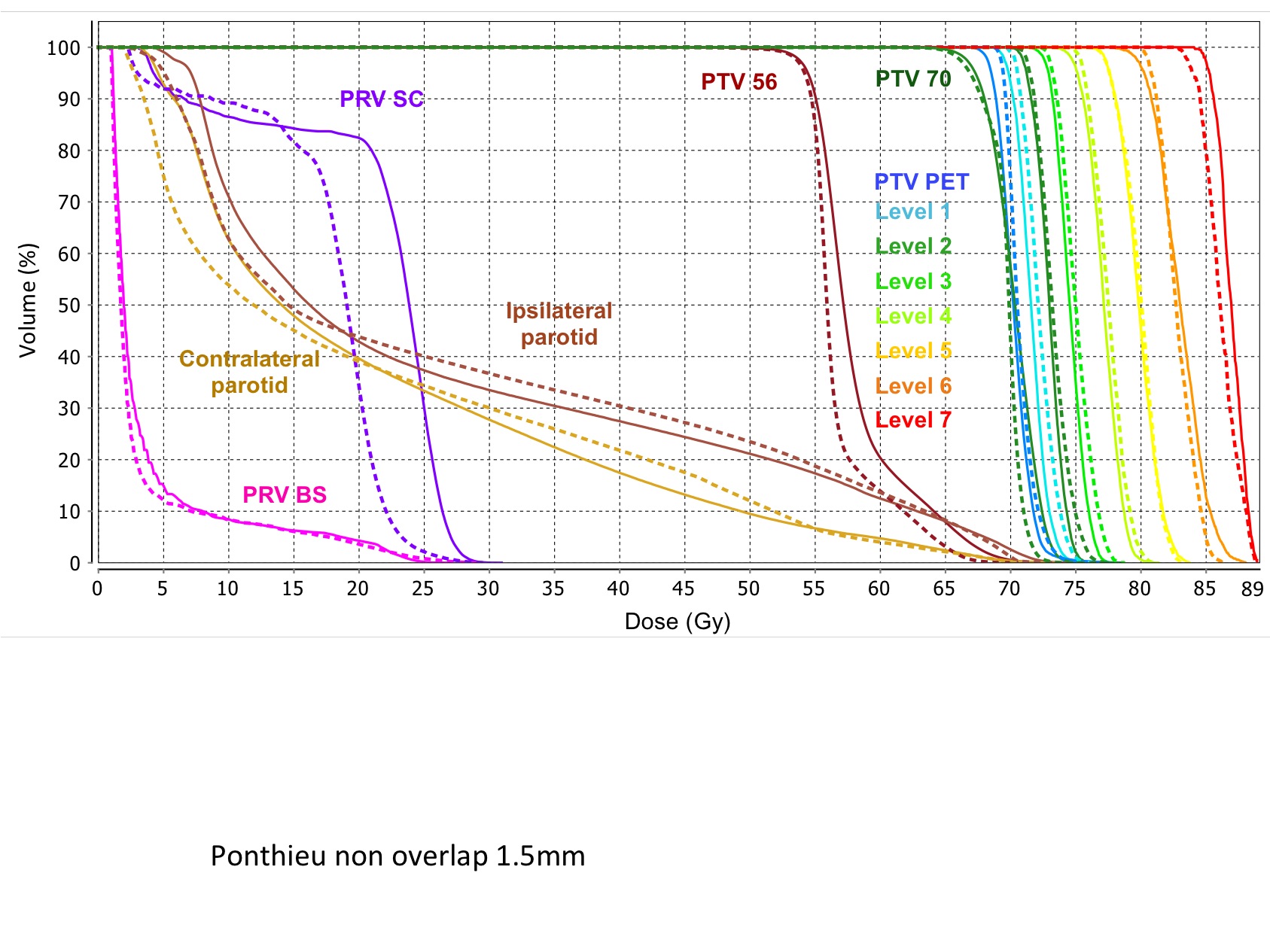


Figure S4. PTV and isodose distribution for Helical Tomotherapy (HT) and Varian RapidArc (RA) for patient #2 for the planning phase II. The captions are zooms of the PTV_PET_.


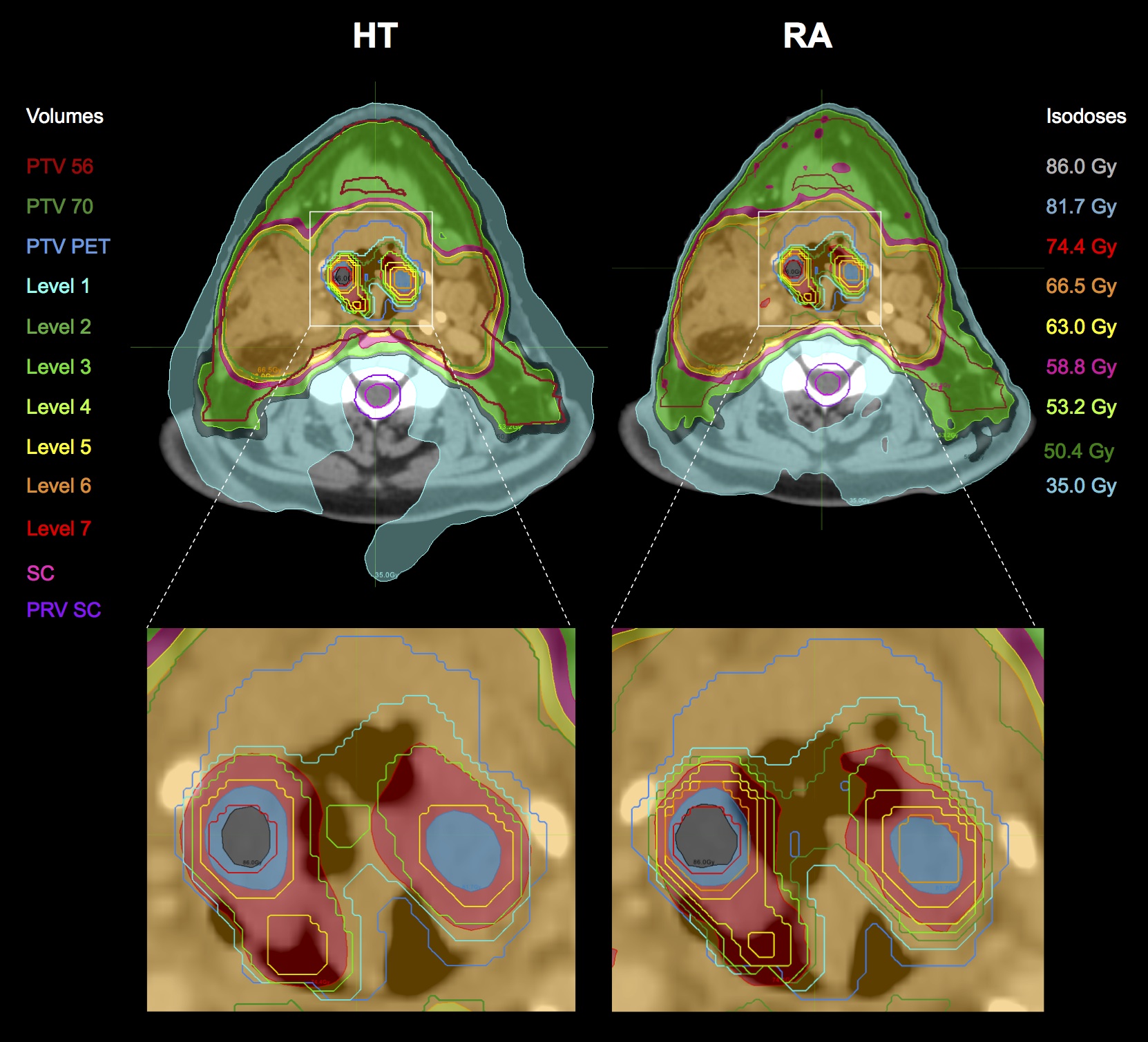


Patient #3

Figure S5. Dose-Volume Histogram (DVH) for the Planning Target Volume (PTV) and the Organ At Risk volume (OAR) for patient #3 for the planning phase II. The DVH for the PTVs are represented in a non-overlapping mode. The plain lines are for RapidArc and the dashed lines are for Helical Tomotherapy. (BS: brain stem; SC: spinal cord).


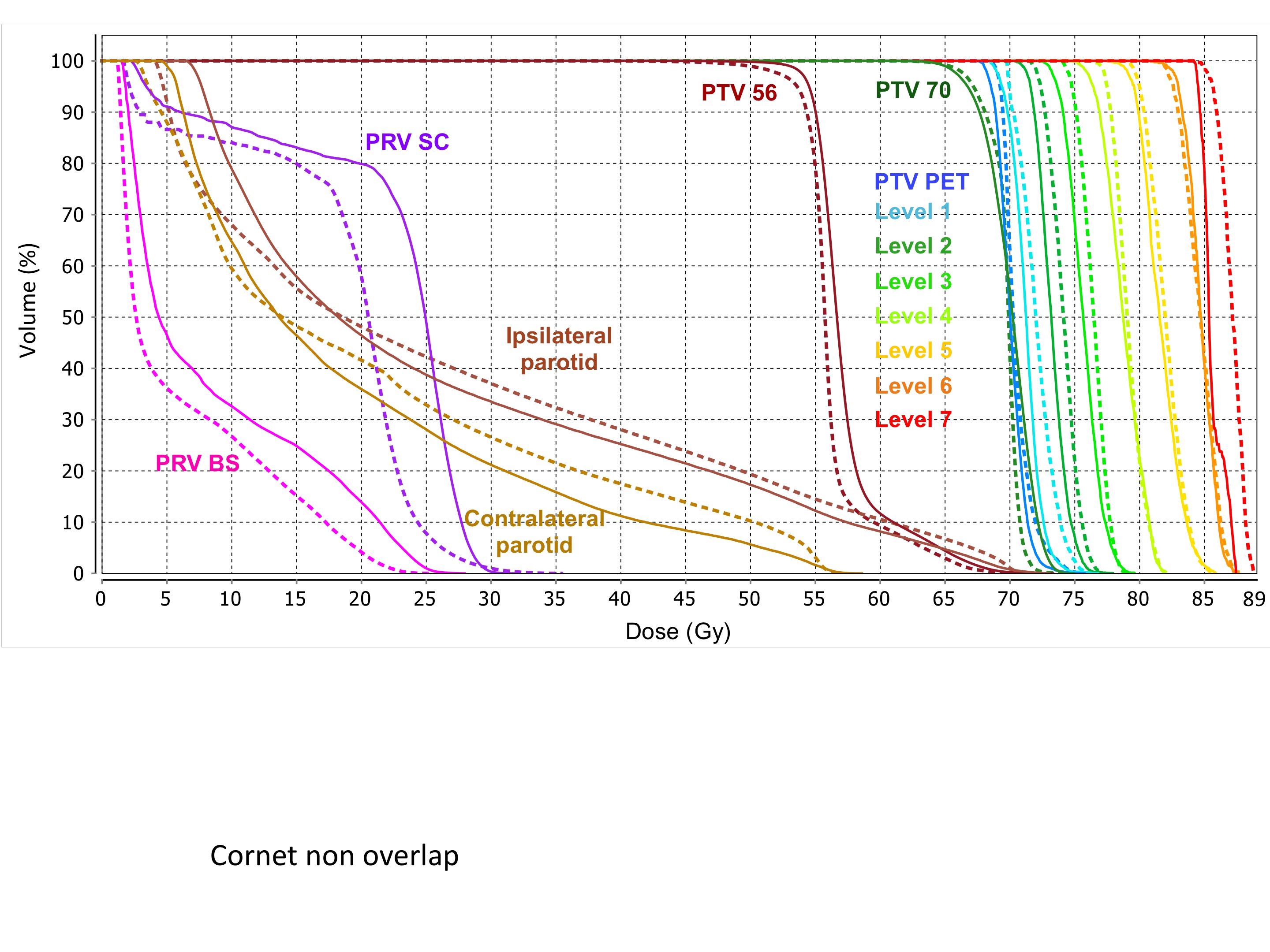


Figure S6. PTV and isodose distribution for Helical Tomotherapy (HT) and Varian RapidArc (RA) for patient #3 for the planning phase II. The captions are zooms of the PTV_PET_.


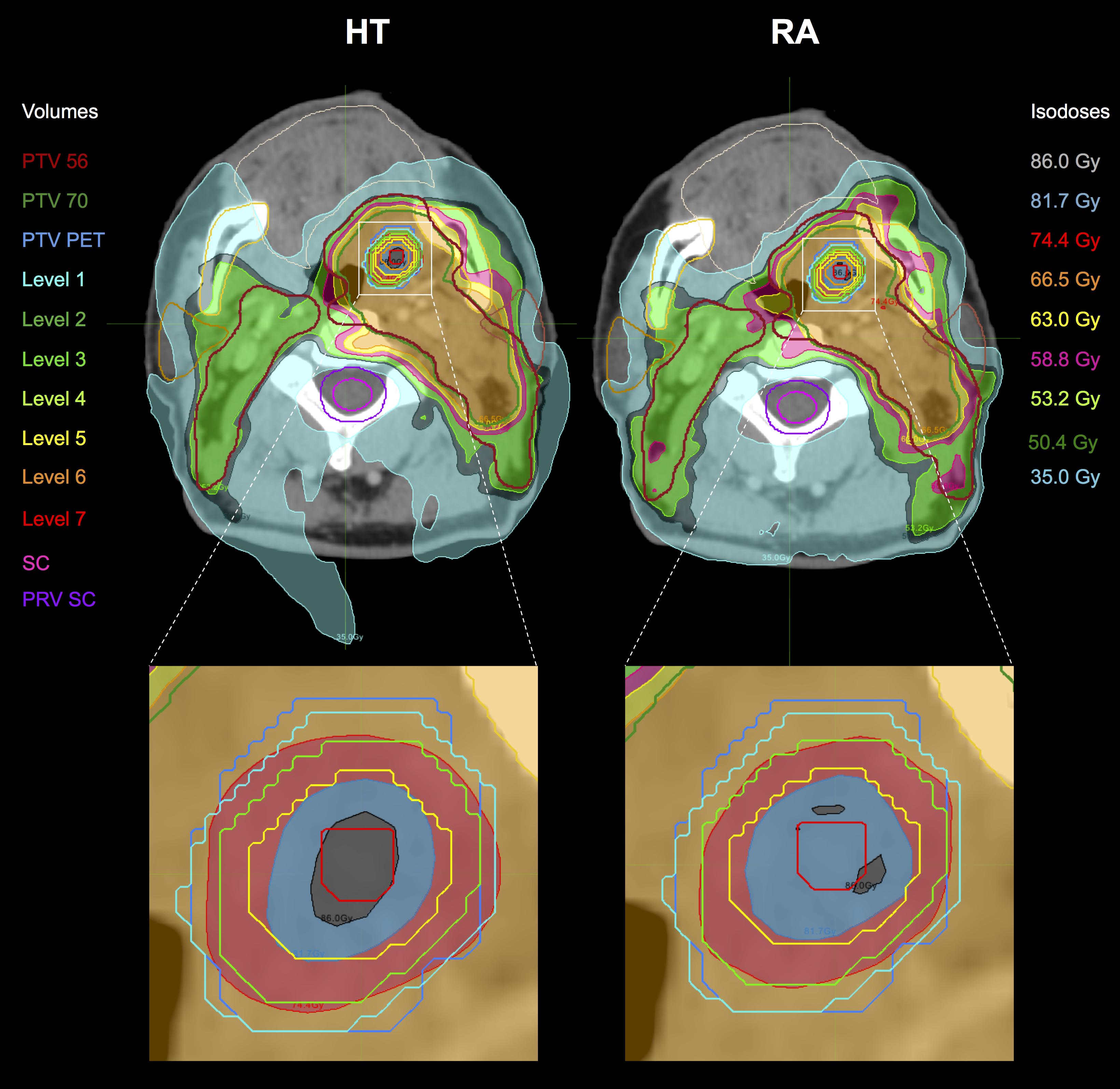


Patient #4

Figure S7. Dose-Volume Histogram (DVH) for the Planning Target Volume (PTV) and the Organ At Risk volume (OAR) for patient #4 for the planning phase II. The DVH for the PTVs are represented in a non-overlapping mode. The plain lines are for RapidArc and the dashed lines are for Helical Tomotherapy. (BS: brain stem; SC: spinal cord).


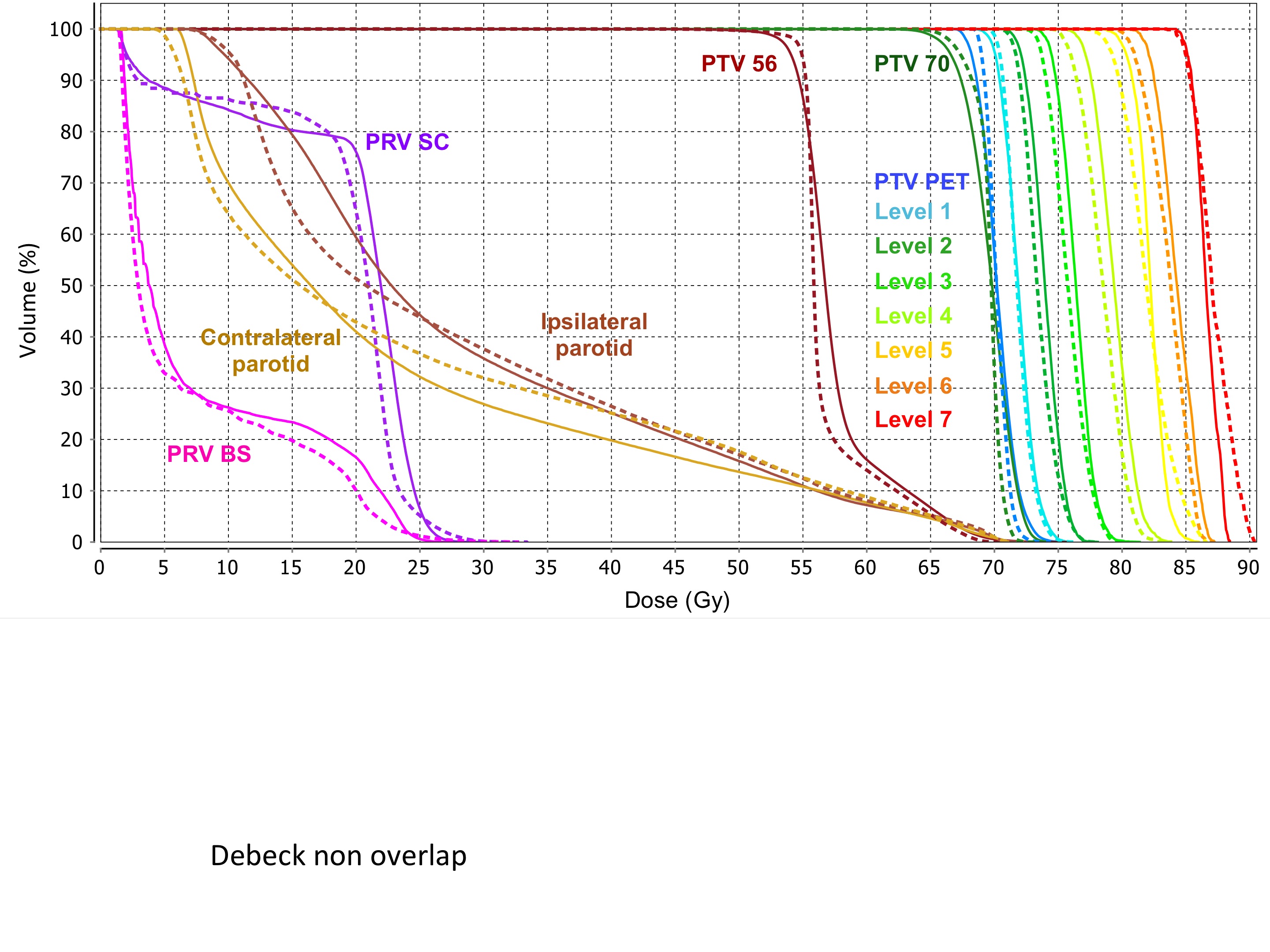


Figure S8. PTV and isodose distribution for Helical Tomotherapy (HT) and Varian RapidArc (RA) for patient #4 for the planning phase II. The captions are zooms of the PTV_PET_.


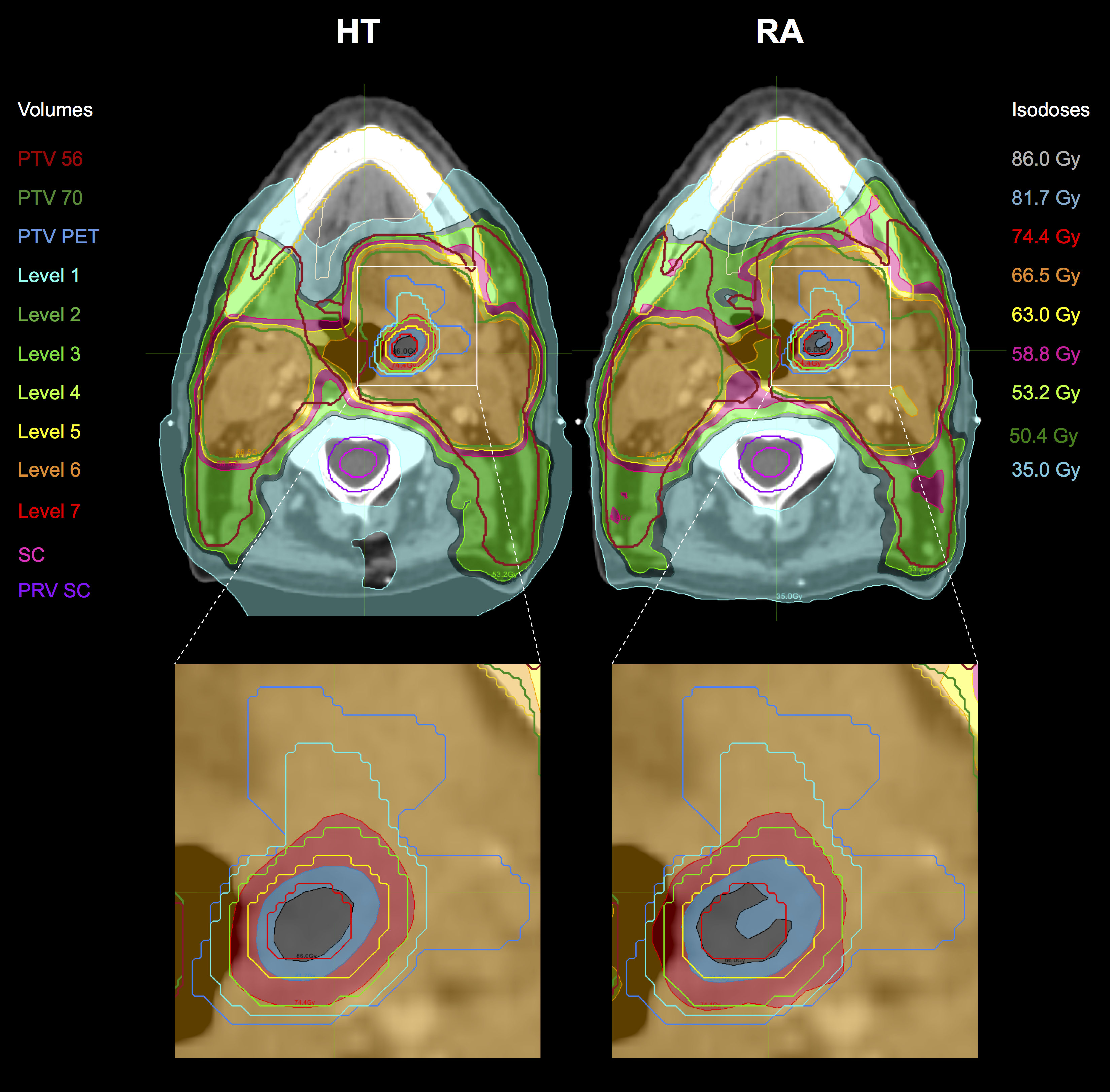

Supplement: Supplementary file 2 — images of individual dose volume histograms and dose distributions of patients. (DOCX 15022 kb) [file 13014_2017_793_MOESM2_ESM.docx]
