# Supplementary material for: Radiation dose escalation based on FDG-PET driven dose painting by numbers in oropharyngeal squamous cell carcinoma: a dosimetric comparison between TomoTherapy-HA and RapidArc
Source: Radiat Oncol. 2017 Mar 23;12:59. doi: 10.1186/s13014-017-0793-0 (PMC5364636; doi:10.1186/s13014-017-0793-0)
Supplement: Supplementary file 3 — Quality volume histograms of the five patients for planning phase I. (DOCX 116 kb) [file 13014_2017_793_MOESM3_ESM.docx]

Additional file 3: quality volume histograms of the five patients for planning phase I

Figure S1. Quality Volume Histogram (QVH) for the PTV_PET_ for patient #1.

Figure S2. Quality Volume Histogram (QVH) for the PTV_PET_ for patient #2.

Figure S3. Quality Volume Histogram (QVH) for the PTV_PET_ for patient #3

Figure S4. Quality Volume Histogram (QVH) for the PTV_PET_ for patient #4.
