# Supplementary material for: Radiation dose escalation based on FDG-PET driven dose painting by numbers in oropharyngeal squamous cell carcinoma: a dosimetric comparison between TomoTherapy-HA and RapidArc
Source: Radiat Oncol. 2017 Mar 23;12:59. doi: 10.1186/s13014-017-0793-0 (PMC5364636; doi:10.1186/s13014-017-0793-0)
Supplement: Supplementary file 4 — Delivery quality assurance for patient # 3. (DOCX 20 kb) [file 13014_2017_793_MOESM4_ESM.docx]

Additional file 4: Delivery quality assurance for patient # 3

Two DQA with different configurations were performed for each technique.

Helical Tomotherapy:

In the first DQA, the film was placed in a sagittal plane passing through the 7^th^ level of the dose painting volume to check the capability of the machine to deliver accurately the gradients close to the highest prescription dose. Three ion chambers (IC) were placed in the transverse plane in homogeneous regions (IC 1: in the elective nodal PTV_56Gy_, IC 2&3: in the therapeutic nodal PTV_70Gy_).

In the second DQA, the film was placed in a sagittal plane through the elective nodal PTV_56Gy_ while ICs were placed in a transverse plane passing through the dose painting volume (IC 1: centered on level 7 of the dose painting volume, IC 2&3: next to chamber 1 in the gradient of the PTV_70Gy_). The position of the first chamber was selected to quantify the accuracy in the painted volume using ion chamber, which is more reliable for local absolute dose measurements.

The gamma index was calculated for global normalization within a selected region-of-interest, namely a rectangle encompassing the area within 5 – 10 mm from the film edges.

RapidArc:

In the first DQA, the film was placed in a coronal plane passing through the 7^th^ level of the dose painting volume. The IC was placed 2 cm posteriorly in a homogeneous PTV_70Gy_ region.

In the second DQA, the film was placed in a sagittal plane passing through the 7^th^ level of the dose painting volume. The IC was placed 3 cm on the right in a homogeneous PTV_70Gy_ region.

The gamma index was calculated for global normalization with dose thresholds of 10% and 50%.

The results are summarized in the following table.

|  | HT | | RA | |
| --- | --- | --- | --- | --- |
|  | DQA 1 | DQA 2 | DQA 1 | DQA 2 |
| Film dosimetry^a^ | 98.9% | 99.7% | 99.0% / 99.8% ^c^ | 96.2% / 98.3% ^c^ |
| Ionization chamber 1^b^ | + 0.3% | + 0.7% | + 2.3% | + 2.5% |
| Ionization chamber 2 ^b^ | - 0.4% | - 1.4% | - | - |
| Ionization chamber 3 ^b^ | - 0.2% | + 1.0% | - | - |

^a^ percentage of pixels passing the gamma-criteria of 3%/3 mm

^b^ discrepancy between planned and measured dose

^c^ 10% and 50% dose threshold respectively
